# Role of Computer-Assisted Surgery in the Management of Pediatric Orbital Tumors: Insights from a Leading Referral Center

**DOI:** 10.3390/children12121649

**Published:** 2025-12-04

**Authors:** Elena Gomez Garcia, Maria Granados, Javier M. Saceda, Maria N. Moreno, Jorge Zamorano, Jose L. Cebrian, Susana Noval

**Affiliations:** 1 IdiPaz, Oral and Maxillofacial Surgery Department, La Paz University Hospital, 28046 Madrid, Spain; elenam.gomez@salud.madrid.org; 2 Department of Ophthalmology, La Paz University Hospital, 28046 Madrid, Spain; 3 Department of Paediatric Neurosurgery, La Paz University Hospital, 28046 Madrid, Spain; 4 IdiPaz, Service of Oral and Maxillofacial Surgery Service, La Paz UnivesityHospital, 28046 Madrid, Spain; 5 IdiPaz, Department of Pediatric Ophthalmology, La Paz University Hospital, 28046 Madrid, Spain; susana.noval@salud.madrid.org

**Keywords:** pediatric orbital tumors, benign, malignant, intraoperative navigation, surgical 3d planning, biomodels, IPS, computed-assisted surgery (CAS)

## Abstract

**Highlights:**

**What are the main findings?**

- Pediatric orbital tumors.
- 3D planning and innovative computer-aided technologies

**What is the implication of the main finding?**

- Multidisciplinary treatment
- Usefulness of Computed-Assisted surgery in pediatric orbital tumors

**Abstract:**

Background/Objectives: Pediatric orbital tumors are rare and complex, requiring multidisciplinary care at specialized centers. Contemporary treatment paradigms emphasize centralized care delivery through experienced multidisciplinary teams to optimize patient outcomes. Recent advances in surgical planning technologies and intraoperative navigation systems have substantially enhanced surgical safety through improvement in tumor resection and reconstruction and reduction in complications, including recurrence of the lesion. Computed-aided surgical technologies enable precise virtual planning, minimally invasive approaches and more precise reconstruction methods when necessary by mean of patient-specific cutting guides, premolded orbital plates or individual patient solutions (IPS) prosthesis. Three-dimensional biomodelling visualizes tumor architecture and aids localization while preserving neurovascular structures, and real-time neuronavigation improves safety and efficacy. Methods: We conducted a retrospective analysis of 98 pediatric patients with orbital tumors treated between 2014 and 2025 at a tertiary center to evaluate the use of computed-assisted surgical technologies and the indications for treatment. Inclusion criteria comprised all cases where computer-assisted techniques were employed. Patients were classified into two groups: Group 1—intraconal or extensive periorbital lesions with eye-sparing intent treated via craniofacial approaches; Group 2—periorbital tumors with orbital wall involvement, to analyze the use of the different technologies. Data collected included tumor age, type, location, technology used, adjunctive treatments, and postoperative outcomes. Results: Twelve patients underwent computer-assisted surgery. Technologies employed over the last six years included intraoperative navigation, 3D planning with/without tumor segmentation, orbital-wall reconstruction by mirroring, IPS or titanium mesh bending, and preoperative biomodelling. Patients were grouped by tumor location and treatment goals: Group 1—intraorbital lesions (primarily intraconal or 270–360° involvement), including one case of orbital encephalocele treated transcranially; Group 2—periorbital tumors with orbital-wall destruction, treated mainly via midfacial approaches. Intraoperative navigation was used in 10/12 cases (8/11 with tumor segmentation); in 3 cases with ill-defined margins, navigation localized residual tumor. Virtual surgery predominated in Group 2 (4 patients) and one in Group 1, combined with cutting guides for margins and Individual Prosthetic Solutions (IPS) prosthesis fitting (two patients: titanium and PEEK). In two cases, virtual plans were performed, STL models printed, and premolded titanium meshes used. No complications related to tumor persistence or orbital disturbance were observed. Conclusions: Advanced surgical technologies substantially enhance safety, efficiency, and outcomes in pediatric orbital tumors. Technology-assisted approaches represent a paradigm shift in this complex field. Additional studies are needed to establish evidence-based protocols for systematic integration of technology in pediatric orbital tumor management.

## 1. Introduction

Orbital tumors in the pediatric population represent a rare and complex clinical entity. Their low incidence and the absence of large patient series in the literature pose significant challenges to establishing standardized treatment protocols [1,2,3]. As a result, the management of these tumors has progressively shifted toward specialized tertiary referral centers, where it is possible to conduct prospective and retrospective studies addressing tumor classification, histopathology, and therapeutic strategies. Moreover, this centralization fosters the development of multidisciplinary teams that integrate cutting-edge medical and surgical modalities to optimize patient care.

In recent years, computer-assisted surgery (CAS) has emerged as a valuable tool in craniofacial procedures, extending beyond its initial application in neurosurgery. Techniques such as intraoperative navigation, virtual surgical planning with tumor segmentation, the use of stereolithographic (STL) models for mesh pre-contouring, and the development of individual patient solutions (IPS) have gained increasing relevance in the cranio-maxillofacial field. In the context of orbital surgery, CAS has primarily been utilized in the management of orbital fractures, with some reports describing its use in orbital decompression for Graves’ disease and selected tumor cases [4,5,6]. However, to date, there are no published studies specifically addressing the use of CAS in the surgical management of pediatric orbital tumors.

This subset of orbital pathology presents unique challenges, including a limited surgical field, the need for meticulous preservation of vital anatomical structures, and consideration of future craniofacial growth. In this context, the implementation of advanced, image-guided surgical technologies aims to improve surgical precision and safety. Additionally, the growing emphasis on globe-sparing procedures and the demand for accurate orbital reconstruction to preserve ocular function have further justified the integration of CAS. These technologies offer the potential to enhance intraoperative decision-making, reduce operative time, minimize complications, and ultimately improve postoperative outcomes in this vulnerable patient population.

## 2. Materials and Methods

Gen IA has been used in this paper to generate text and refine English style.

### 2.1. Study Design and Setting

This retrospective observational study was conducted at the La Paz University Hospital, a tertiary care center in Madrid, Spain, within the Orbit Tumor Reference Unit. The study reviewed medical records of patients treated between 2014 and 2025. The institutional review board approved the study protocol, and all procedures complied with relevant ethical standards and patient confidentiality requirements.

### 2.2. Patient Population

We screened all patients treated at the Orbital Tumors Reference Unit during the study period. A total of 98 patients were identified and included in the analysis, encompassing all orbital tumor types. Of these, 64 patients had benign tumors and 34 had malignant tumors. All patients were operated on by the same multidisciplinary surgical team.

### 2.3. Inclusion Criteria

From the reviewed cohort, we selected all patients who underwent surgical treatment via a craniofacial approach for tumors located at the orbital apex (cone orbitale) or tumors affecting 270–360° of the orbital volume with an eye-sparing surgery and patients with tumors affecting the orbital boundaries with the need for orbital wall reconstruction. Importantly, the cohort included the use of computer-assisted surgery (CAS) techniques in the perioperative or intraoperative workflow, including the following:

- Intraoperative navigation using STEALTHSTATION S8 SURGICAL NAVIGATION SYSTEM
- Preoperative planning and segmentation with delineation of the tumor
- Mirroring for mirrored anatomy or planning
- Printing of models for implant shaping or fabrication of IPS prostheses
- Design and printing of anatomical biomodels including the tumor to guide intraoperative access routes

Both benign and malignant tumors meeting these anatomical and technological criteria were included. None of the patients involved enucleation or orbital exenteration in the surgical plan (eye-sparing surgery).

### 2.4. Exclusion Criteria

Patients lacking complete surgical or imaging records, or those treated with non-craniofacial approaches that did not involve the specified orbital volume, were excluded from the surgical-subgroup analysis.

### 2.5. Data Collection

Data were abstracted from electronic medical records and surgical reports, including demographics, tumor histology [benign vs. malignant, histopathological type], tumor location, extent of orbital involvement (specifically 270–360°), surgical approach details, intraoperative findings, extent of resection, reconstruction methods, perioperative complications, and short-term and long-term outcomes. Imaging studies used for eligibility and assessment included preoperative MRI/CT scans and, when available, postoperative imaging. Details of CAS modalities, navigation systems, planning software, segmentation outputs, and 3D-printed models/prostheses were recorded where available.

### 2.6. Outcome Measures

Primary outcome: feasibility and safety of craniofacial approaches for tumors involving 270–360° of the orbital volume or those located in the intraconal space, in the context of computer-assisted surgical workflows.

Secondary outcomes: extent of resection, perioperative complication rate, accuracy of tumor delineation, and early functional/anatomical outcomes related to CAS-guided procedures.

### 2.7. Statistical Analysis

Descriptive statistics were used to summarize patient characteristics, tumor types, CAS utilization, and outcomes. No formal comparative statistical test was planned for this descriptive retrospective study unless specified otherwise in the protocol. Due to the small sample size and the diversity of lesions included in the study, no comparative analysis of conventional treatments was conducted. Instead, the descriptive assessment of the clinical applications of the various computer-assisted surgery techniques was performed to evaluate their utility.

### 2.8. Note on Terminology

The term “craniofacial approach” refers to surgical corridors that combine skull base craniotomy with orbital exposure, enabling access to tumors at the orbital apex or involving substantial orbital volume. The designation “270–360° of orbital volume” denotes involvement or encasement of most of the orbital circumference, as assessed by preoperative imaging and intraoperative judgment. “Computer-assisted surgery” includes intraoperative navigation, preoperative planning and segmentation, mirroring, and the production of 3D-printed models for implant shaping or IPS prosthesis fabrication, as well as biomodel printing that incorporates the tumor for planning intraoperative access routes.

## 3. Results

Globe-sparing surgery was achieved in all treated patients, as previously noted. All patients were operated on by the same multidisciplinary surgical team.

A review was conducted of orbital tumors or tumors involving the orbit treated with computer-assisted surgery [CAS]. Of 98 cases analyzed in our series from 2014 to 2025, 12 employed CAS tools. Preoperative evaluation included multidisciplinary review by the Orbital Tumor Committee and the Oncology Committee of Hospital Universitario La Paz. Surgeries were performed by a multidisciplinary craniofacial team comprising a pediatric neurosurgeon, a pediatric orbital ophthalmologist, and a pediatric maxillofacial surgeon.

The earliest CAS-treated case was in 2019; subsequent cases were treated from 2020 to 2025, with distribution summarized in Table 1.

**Figure 1:**
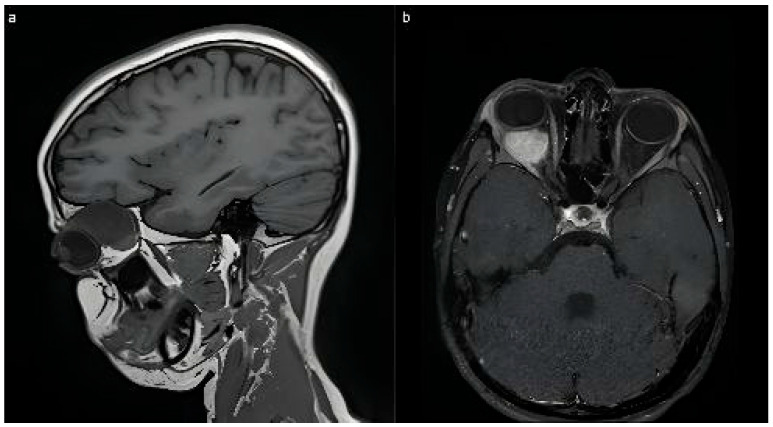
MRI image of an Intraconal tumor affecting the right eye. Note the extension of the lesion in the orbital apex and the clear margins of the tumor. (**a**) sagittal; (**b**)axial view.

**Figure 2:**
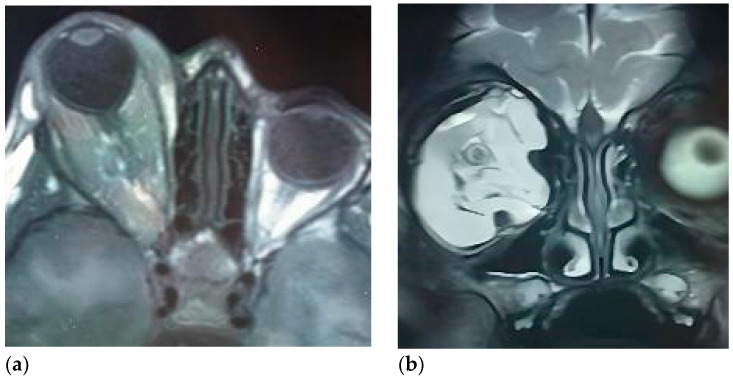
MRI image of the lymphatic malformation affecting almost 360° of the orbital compartment. (**a**) axial view; (**b**) coronal view.

**Figure 3:**
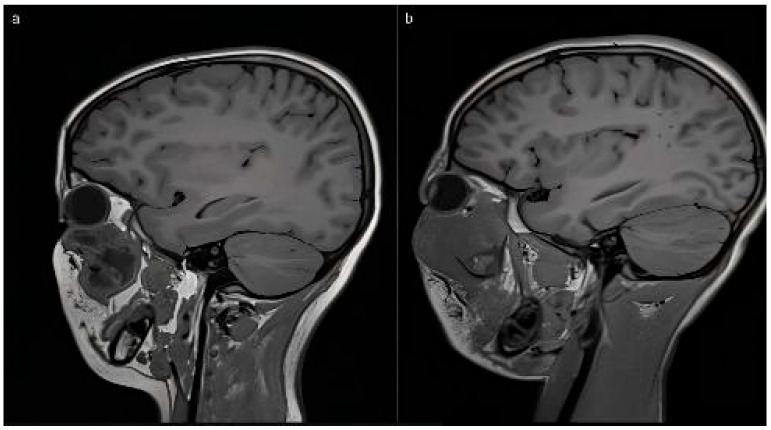
MRI images from both induced osteosarcomas affecting the maxillary sinus region (**a**) and the extent to the posterior orbital region and the pterygopalatine areas (**b**).

CAS-treated patients were categorized into two major groups, irrespective of malignancy status, with eye-sparing protocols applied in all cases:

(1) Intraorbital tumors (Group 1): lesions primarily involving the intraconal space at the orbital apex or extending across multiple orbital compartments (approximately 270–360°). This group included one patient with intracranial communication due to an orbital encephalocele. Surgical approach was transcranial via coronal or extended hemicoronal incisions, harvesting a galea-pericranial flap, fronto-orbital craniotomy with extension toward the pterional region according to lesion location, and a supraorbital bar extending laterally to the supraorbital nerve, with the orbital roof addressed in one to two pieces (Figure 1 and Figure 2).
(2) Tumors originating from periorbital structures with destruction of orbital walls (floor, medial wall, or orbital roof) (Group 2) (Figure 3).

**Figure 4:**
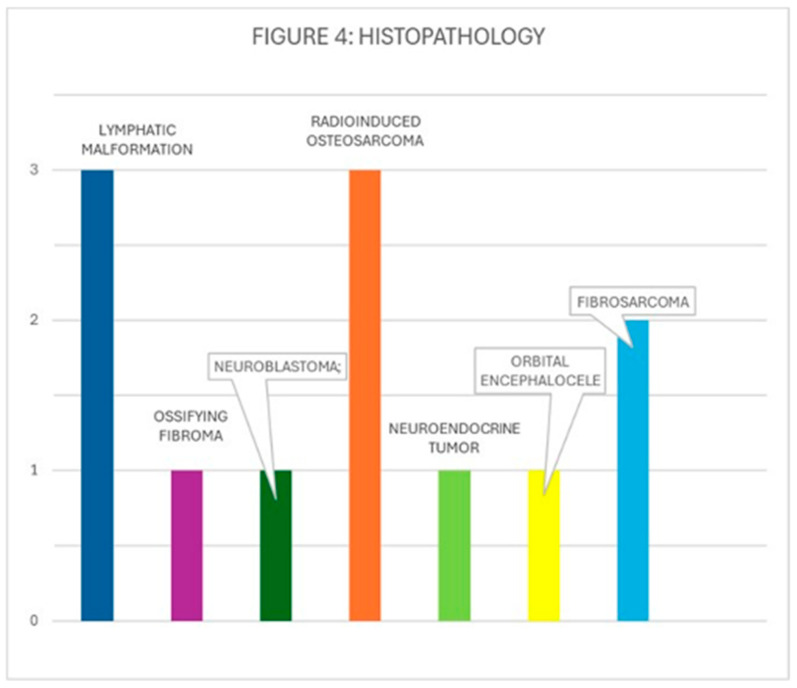
Histopathology distribution.

Histological distribution (Figure 4)

− Group 1 [intraorbital tumors]: fibrosarcoma (2), orbital encephalocele (1), intraorbital lymphatic or lymphatic-venous malformation (3), neuroendocrine tumor (1). Poorly differentiated neuroblastoma with a low mitosis index (1)
− Group 2: radiation-induced osteosarcoma (3), fibro-osseous lesion/osteogenic tumor (1).

Figure 4 histological distribution of the treated tumors.

**Figure 5:**
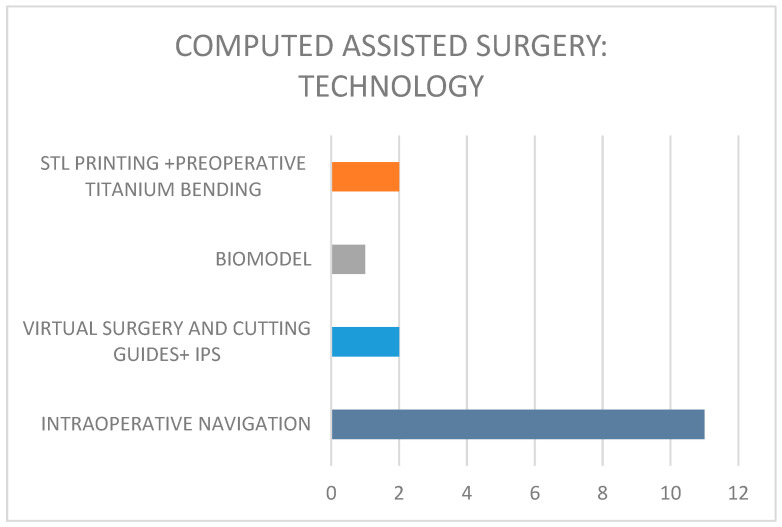
CAS technologies used in the reported series.

CAS techniques employed (Figure 5)

− Lesion segmentation and intraoperative navigation (*n* = 11) using the STEALTHSTATION S8 (Medtronic).
− Virtual surgery with biomodel design (*n* = 1).
− Virtual surgery with mirroring, STL printing, and in-house titanium mesh customization (*n* = 2).
− Virtual surgery with mirroring and IPS planning, including cutting guides (*n* = 2). Protheses fabricated from PEEK (*n* = 1) and titanium ((*n* = 1).

Figure 5: Computed-Assisted Techniques used in our series.

### 3.1. Treatment and Follow-Up

All reviewed patients underwent surgical intervention. For malignant cases, treatment followed the oncology committee’s tumor protocol at the national sarcoma reference center. Preoperative and postoperative adjuvant therapies [chemotherapy, radiotherapy, or proton therapy] were tailored individually.

### 3.2. Secondary Results

**Figure 6:**
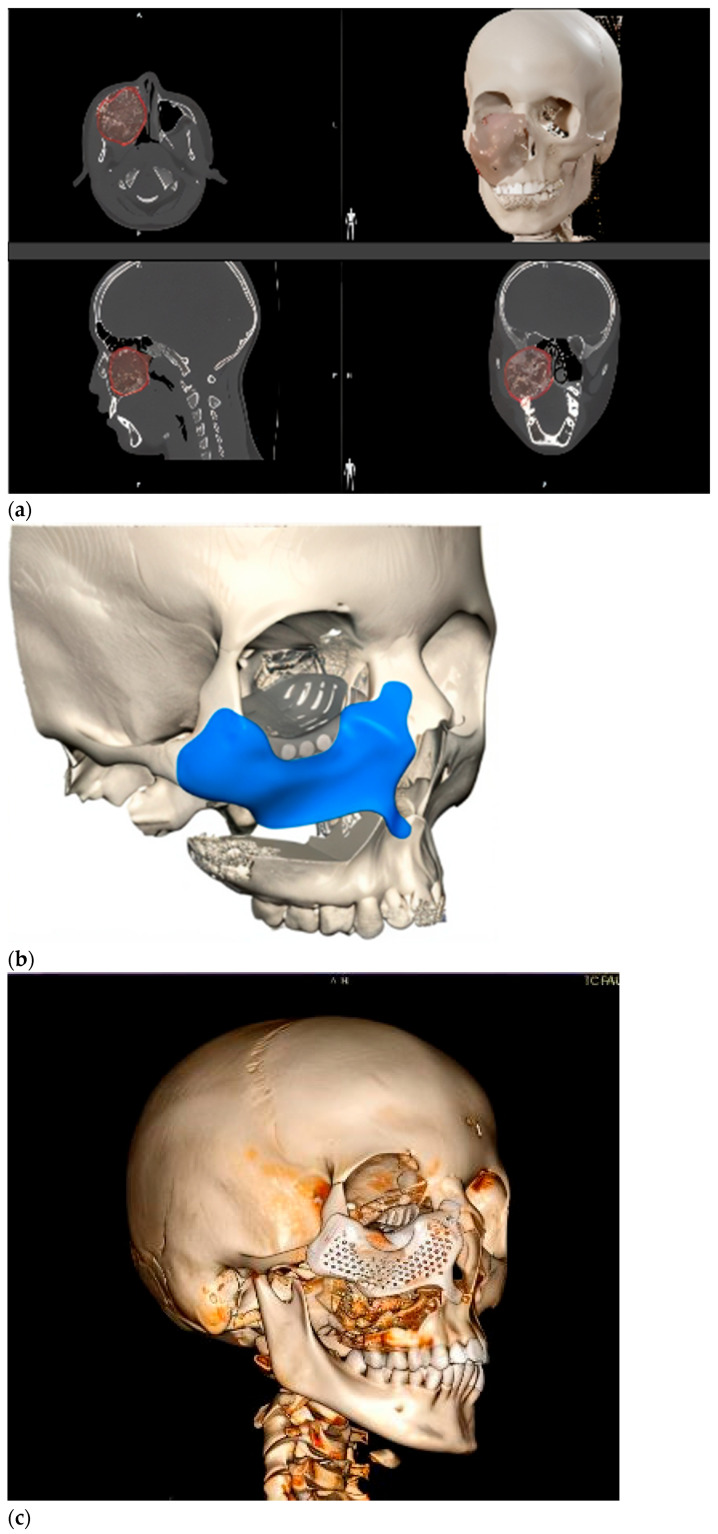
Ossifying fibroma affecting right maxilla and orbit causing orbital dystopia. (**a**) Tumor segmentation prior to surgery allows intraoperative navigation to control tumor margins and proper IPS positioning. (**b**) Virtual IPS design after virtual tumor resection was achieved, in this case using a titanium structure to minimize infection risk. The two-piece design improves proper positioning under smaller approaches. Note a blue structure corresponding to the infraorbital rim and malar bone and a grey structure to replace the orbital floor. Both pieces are connected by means of titanium screws. (**c**) Postoperative scan with the IPS in place.

**Figure 7:**
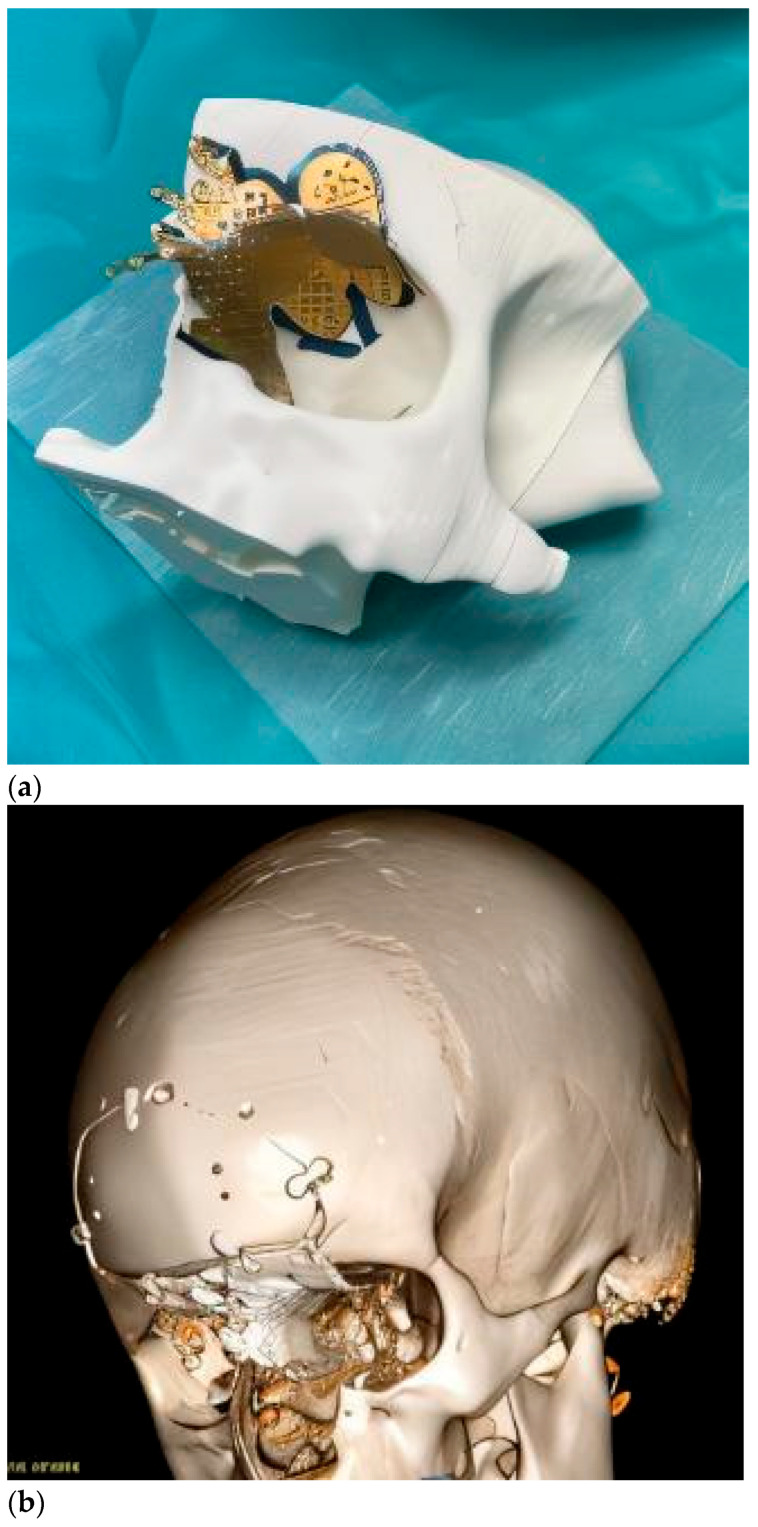
Virtual surgery with posterior orbital mirroring for plate bending was employed in this patient. (**a**) For the operating room, a new orbital STL model (mirror of the healthy orbit) was printed, and a commercial mesh was pre-molded for use during surgery, enabling a pre-planned virtual procedure with reduced costs. The printed STL model with the titanium mesh adapted to the defect. (**b**) postoperative scan with the surgical result. This approach is limited by the potential need for unplanned extended resections, during which the available reconstructive mesh may be insufficient.

**Figure 8:**
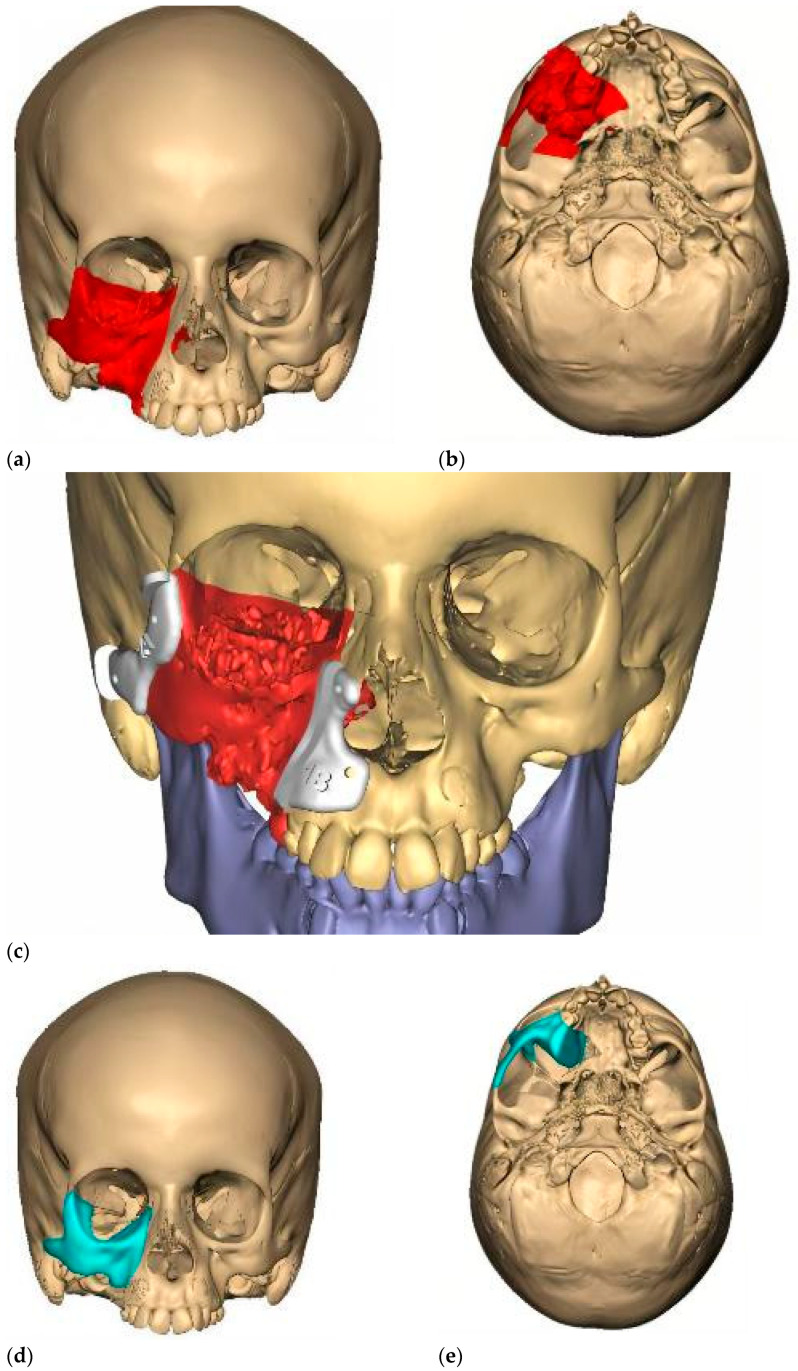
(**a**) Virtual surgical plan for a radio-induced osteosarcoma, including resection margins in frontal view. The decided surgical margins are delineated in red. (**b**) Inferior view of the virtual resection plan with margins, coloured in red. (**c**) Design of cutting guides in white; these guides translate the information from the resection margins to the surgical field. The mandible is coloured in purple. (**d**) Frontal view of the IPS (PEEK prosthesis) design, coloured in blue. (**e**) Inferior view of the IPS design reconstructing the malar bone, infraorbital rim, and zygomatic arch structures, coloured in blue.

Intraoperative navigation was the most widely used CAS modality in both groups. For tumors with extraorbital extension (Group 2), navigation was combined with virtual mirroring, IPS planning, and cutting guides (notably in fibro-osseous lesions) (Figure 6). The remaining two osteosarcoma cases involved virtual mirroring and titanium plate modeling on the STL, in conjunction with prior lesion segmentation and intraoperative navigation (Figure 7). In one maxillary osteosarcoma case, navigation was not used; however, virtual planning with a PEEK IPS prosthesis was performed (Figure 8).

For two cases of radiation-induced osteosarcoma with complex lesion location, patient-specific models were produced to plan the approach and understand anatomical relationships (Figure 8).

**Figure 9:**
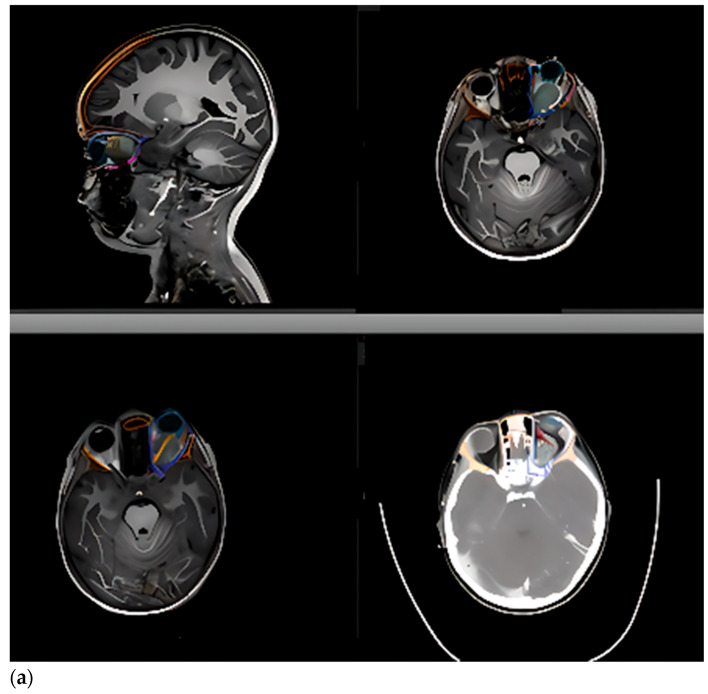

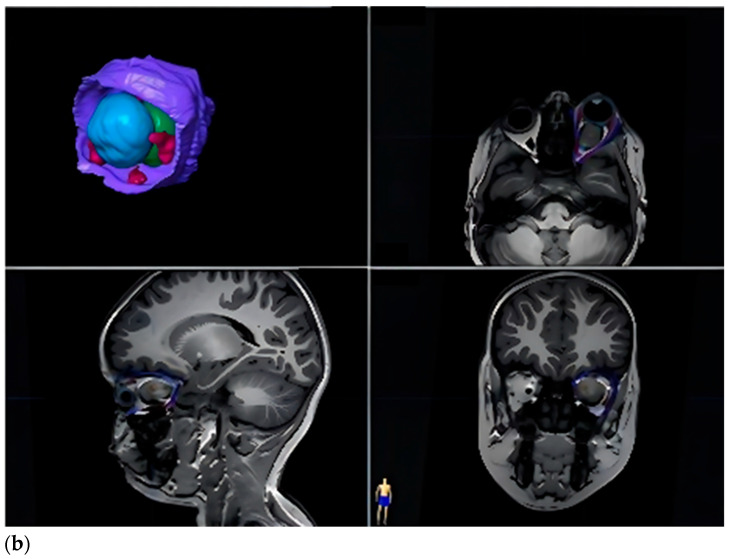
(**a**) Preoperative MRI-CT fusion is essential for navigating intraorbital lesions and accurately identifying soft-tissue **structures (e.g., extraocular** muscles, optic nerve, and tumor). (**b**) Constructed biomodels can be printed after this segmentation to help visualize tumor relationships, plan surgical corridors, and anticipate postoperative outcomes.

Intraorbital tumors (Group 1) consistently used intraoperative navigation. In orbital lymphatic malformations, preoperative lesion segmentation was not performed due to delineation difficulties; navigation relied on MRI to delineate soft tissue structures (optic nerve and extraocular muscles) (Figure 9).

In one intraorbital fibrosarcoma, lesion segmentation, biomodel printing, a supraorbital approach cutting guide, and intraoperative navigation were implemented.

**Figure 10:**
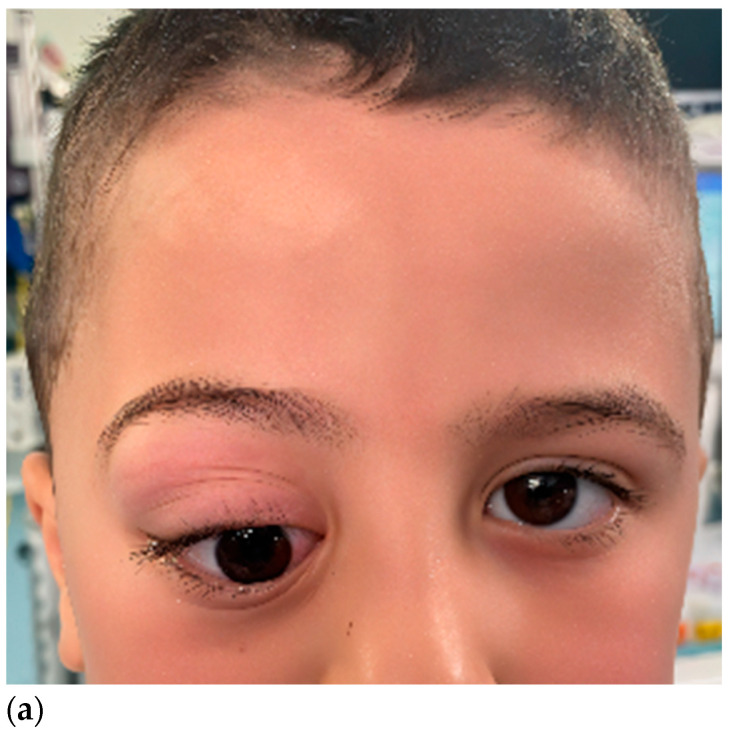

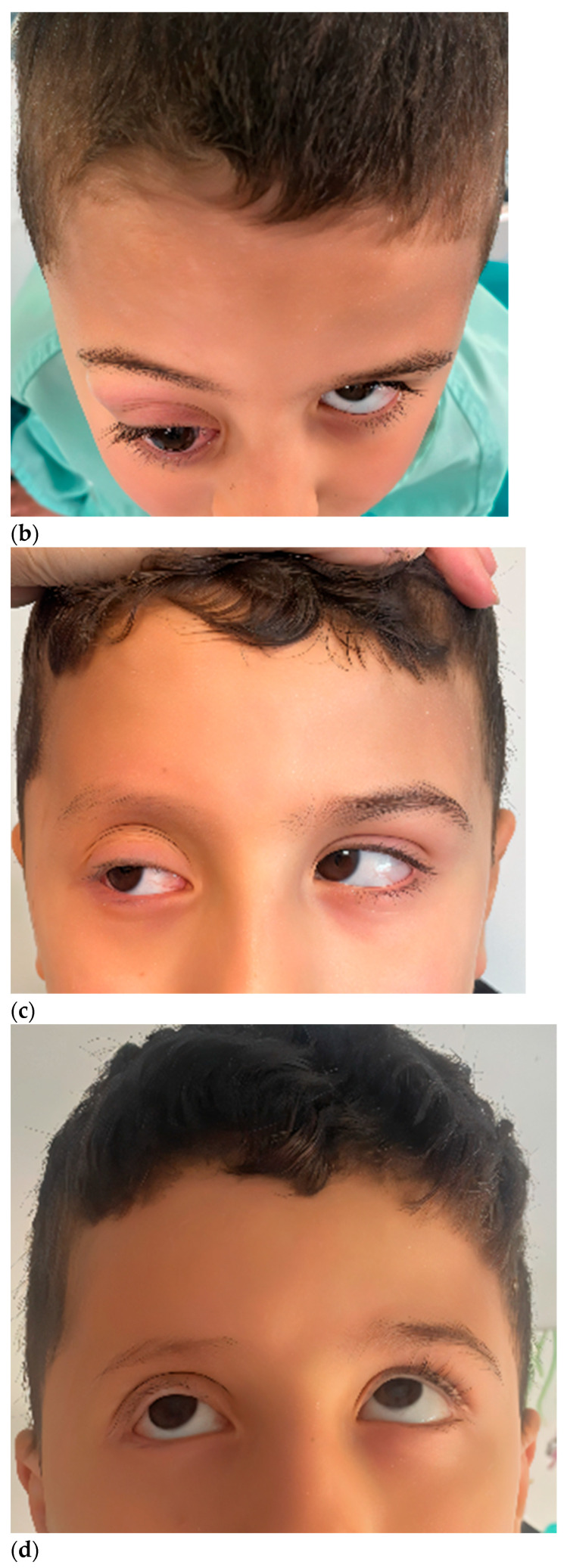

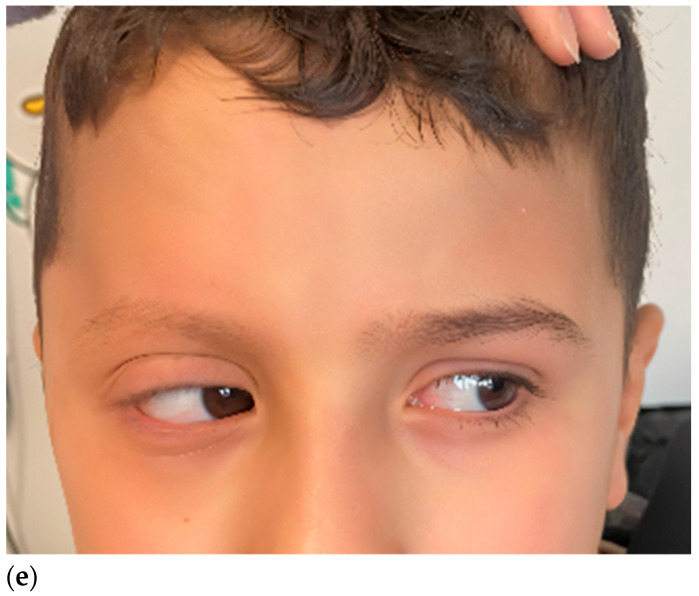
Clinical view of a patient with intraconal lesion (fibrosarcoma). Preoperative views where orbital dystopia and exophthalmos could be assessed. (**a**,**b**). Postoperative views with good clinical outcome, especially in terms of globe position, and extraocular movements (**c**–**e**).

Overall, intraoperative navigation emerged as the predominant modality, supporting precise localization relative to the globe and critical neurovascular structures, delineation of margins for resection, and verification of implant or mesh positioning relative to orbital reconstruction. However, given substantial alterations in orbital volume and surrounding structures, postoperative globe position relative to contralateral eye symmetry was not a primary determinant of success (Figure 10).

### 3.3. Limitations and Considerations

− Pediatric applications of virtual planning and patient-specific IPS/prosthesis design are constrained by growth considerations and fixation methods, given ongoing orbital development (notably in the first five years, when 80–85% of final size occurs; growth may continue at up to ~10% per year) [7]. These factors influence reconstruction choices and necessitate family/patient counseling about future interventions.
− Mirroring-based symmetry may not fully reflect orbital asymmetry resulting from tumor-related deformities or prior resections, which can limit the accuracy of virtual planning in some pediatric cases.

### 3.4. Future Directions

The pediatric orbital tumor surgical landscape is moving toward minimally invasive approaches [e.g., canthal/ciliary region incisions and robotic-assisted surgery]. Practical challenges persist due to instrument dimensions and the small orbital workspace, particularly in pediatric patients [8,9,10].

## 4. Discussion

The integration of computer-assisted surgery (CAS) and adjunctive digital technologies has advanced substantially in the management of pediatric orbital tumors, enabling more precise preoperative planning and safer intraoperative execution [6,11,12,13]. Virtual model-based surgical navigation and multimodal image fusion (CT and MRI) provide detailed anatomical delineation and three-dimensional (3D) resection planning—capabilities that are particularly critical in the pediatric orbit due to proximity to vital structures. Contemporary computer-guided protocols have demonstrated high geometric fidelity for osteotomies and targeted intraorbital lesion excision, with submillimetric execution error [<1mm] and no intraoperative complications reported in recent series, thereby supporting both open and endoscopic approaches and optimizing structural preservation alongside oncologic radicality [14].

Beyond resection planning, CAS has been adopted for reconstruction of orbital and midfacial defects, enabling more precise resections and customized reconstructions that translate into improved functional and aesthetic outcomes. Additional applications include navigation-guided sampling and “tumor mapping,” as well as 3D documentation of tumor margins that can inform more targeted adjuvant therapies [5,15]. In pediatric cohorts, when tumor size and location are favorable, minimally invasive approaches reinforced by navigation and virtual planning have been associated with reduced operative time, perioperative complications [for example, periorbital edema and dural tears], postoperative pain, and length of stay, with concomitant cosmetic benefits [8].

Patient selection remains pivotal. Children with deeply seated, anatomically constrained tumors adjacent to critical structures [optic nerve, skull base, orbital apex] and lesions of small to moderate size derive the most benefit from CAS-enabled workflows [11]. In such cases, 3D planning, navigation, and minimally invasive techniques facilitate precise resections while mitigating complication risk and preserving function and cosmesis [8]. Conversely, very large tumors or those with intracranial extension frequently necessitate more extensive conventional craniofacial approaches; in these scenarios, CAS may contribute primarily to preoperative planning and intraoperative control (for example, trajectory verification or intraoperative imaging to confirm resection and reconstruction accuracy) rather than reducing approach morbidity per se [4,6,16,17]. Notably, trajectory-guided biopsy has particular value for complex locations or when precise sampling is required for histopathological diagnosis [18].

Comparative evidence on long-term functional and oncologic outcomes between CAS-supported and conventional techniques in pediatric orbital tumors remains limited but is growing. Recent studies suggest that technology-guided minimally invasive corridors—incorporating navigation and virtual planning—confer reductions in surgical complications [periorbital edema, dural tears], postoperative pain, and length of stay, with superior cosmetic outcomes, without compromising extent of resection or local disease control in appropriately selected cases [5,8,19]. With respect to visual preservation, CAS may facilitate more precise resections near the optic apparatus and other critical neurovascular structures, contributing to higher eye-salvage rates and improved postoperative visual function when tumor size and location are amenable [20,21]. However, when the optic nerve is directly infiltrated, visual prognosis is determined predominantly by tumor extent and histology rather than the surgical technology employed, as evidenced by pediatric series of optic pathway gliomas and optic nerve gliomas [22]. Regarding local control, CAS has demonstrated resection margins (R0) that are comparable or superior to conventional surgery in orbital and midfacial oncology, aided by accurate intraoperative verification and enhanced documentation of margins, which can streamline adjuvant treatment planning [5,14]. To date, no significant differences have been consistently reported in overall survival or local recurrence in the limited pediatric series available, likely reflecting small sample sizes and relatively short follow-up intervals [8].

Several limitations and challenges temper the generalization of CAS to high-risk pediatric orbital tumors. First, access and exposure can be insufficient for oncological safe resection in very large, multicompartmental, or intracranially extending lesions, even with sophisticated navigation and endoscopic assistance; conventional approaches remain necessary in a subset of cases [23]. Second, reliance on technology and the associated learning curve can constrain uptake, particularly in lower-volume centers; expertise in navigation systems, virtual planning, and endoscopic or transorbital corridors is required to achieve the reported accuracy and safety [16]. Third, costs and availability of advanced hardware, software, maintenance, and 3D manufacturing restrict widespread access, concentrating on these modalities in highly specialized centers [1,2,8]. Fourth, the main limitation of the intraoperative neuro-navigation system is the displacement of non-bony anatomical structures (e.g., bone invasion, significant bleeding, CSF leaks, or soft-tissue shifts). To mitigate this problem, image-updating strategies could be employed, such as ultrasound integrated into the neuronavigational system or the acquisition of real-time updated images using intraoperative CT or MRI [5,14]. Finally, proximity to the optic apparatus, orbital apex, and skull base confers persistent neurological and functional risk, and CAS does not abolish the potential for iatrogenic injury; in selected high-risk scenarios, morbidity may approximate that of conventional approaches despite technological support [14,23].

Taken together, the evidence supports CAS and related technologies as enablers of precise, safer, and often less invasive management of appropriately selected pediatric orbital tumors, with measurable benefits in efficiency, perioperative morbidity, and aesthetic outcomes when anatomical constraints allow. For large or extensively infiltrative tumors, CAS should be leveraged to optimize planning and intraoperative control while recognizing that traditional craniofacial exposures may remain the standard to ensure oncologic completeness. In that group of patients, the aid of virtual surgical planning, associated with presurgical mesh bending or IPS design, are valuable tools to ensure better oncologic, functional and cosmetic outcomes. Future work should prioritize pediatric-specific, prospective evaluations—ideally multicenter—that compare CAS-supported versus conventional strategies on oncologic control, visual function, ocular motility, orbital growth, cosmesis, quality of life, and health economics, alongside continued refinement of minimally invasive and endoscopic corridors and the selective use of intraoperative imaging [8,18,19,21].

CAS relies on high-resolution craniofacial CT DICOM data, complemented by MRI with/without contrast, to delineate tumor extent. Postoperative assessment often includes MRI and a three-month postoperative CT to evaluate concordance between planning and actual anatomy. This framework enables tumor delineation through preoperative segmentation, mirroring for planning intraoperative routes, and the use of biomodels to guide approach selection and reconstructive strategies. In non-oncologic orbital series, navigation-assisted reconstruction shows good agreement with preoperative plans for orbital contour and globe projection, supporting the potential utility of CAS in oncologic orbital surgery when eye preservation is prioritized. Caution is warranted in applying these results to pediatric tumors given growth and developmental considerations [6].

Virtual planning yields 3D-printed models, cutting guides, and patient-specific implants, facilitating precise resections and predictable reconstructions. However, manufacturing these materials is time-consuming and costly, potentially limiting access in resource-constrained settings. Despite these challenges, navigation-guided midfacial reconstruction demonstrates concordance with preoperative plans in selected cases, supporting the evolution toward navigation-mediated craniofacial surgery for complex orbital tumors [4].

Emerging literature describes two forward-looking trajectories: minimally invasive and robotic-assisted orbital surgery. Endoscopic or transorbital approaches may benefit select intraorbital masses by reducing morbidity and improving cosmesis. Robotic systems offer potential for precise resections in constrained spaces near the orbital apex, with theoretical advantages including reduced tissue manipulation and faster recovery. Significant barriers remain, including instrument size, lack of tactile feedback, prolonged setup times, and radiation exposure—particularly pertinent in pediatrics due to smaller orbital dimensions and greater anatomical variability. Dedicated pediatric studies are required to define indications, safety, and cost-effectiveness, and future technology development should focus on pediatric-adapted, smaller platforms and faster production of patient-specific implants [10,13,24,25].

The main clinical implications for pediatric orbital tumor care could be summarized in the following issues:

− CAS-enabled planning, navigation, and reconstruction should be incorporated within multidisciplinary teams at experienced centers, especially for apex-involving tumors or extensive orbital involvement (270–360°).
− Preoperative segmentation, mirroring, and selective use of 3D-printed models or guides may enhance planning and intraoperative decision-making in appropriately selected pediatric cases.
− Given the limited pediatric data, prioritize prospective pediatric studies and registries to evaluate navigation reliability, orbital volume restoration, functional outcomes [vision, ocular motility], cosmesis, and growth trajectories.
− Investigate minimally invasive and robotic approaches in well-selected pediatric cases, with rigorous evaluation of instrument scalability, tactile feedback, setup time, radiation exposure, and cost. Standardized outcome measures will facilitate cross-study comparisons.
− Standardize terminology and metrics to enable meta-analyses and cross-study comparisons (e.g., delineation accuracy, margin status, orbital volume, globe projection, cosmesis, and complication rates).

## 5. Conclusions

Although CAS shows promise for improving planning, intraoperative accuracy, and reconstruction in pediatric orbital tumors, pediatric evidence remains limited. Targeted pediatric research is essential to define indications, optimize outcomes, and ensure safe, cost-effective integration of these technologies into pediatric orbital tumor care.

## 6. Declaration of Generative AI and AI-Assisted Technologies in the Writing Process

During the preparation of this work, AI-based tools (including ChatGPT5.1 and OpenEvidence2025) were used solely for language editing and to refine the scope. All substantive content and references were prepared and verified by the authors. No substantive content was generated by AI.

## Figures and Tables

**Table 1:** Demographic and clinical characteristics of pediatric patients with orbital tumors who underwent computer-assisted surgery (CAS). Data are shown for the cohort included in this study. Variables included age at surgery, sex, tumor histology, laterality, prior treatments, presenting symptoms, imaging findings, CAS modality used, extent of resection, perioperative complications, and follow-up outcomes.

CASE	Age at Diagnosis	Sex	Eye	Presenting Symptom	LOCALIZACION	Year of Treatment	AP	INTRAOPERATIVE NAVEGATION	VIRTUAL SURGERY (CUTTING GUIDES	IPS	STL PRINTING + PREOPERATIVE TITANIUM BENDING	Surgery	CHT	RT	Relapse
1	9	F	RE	Cheek swelling	Extension to the orbital floor from maxilla	2019	radioinduced osteosarcoma	NO	YES	YES	NO	Maxilla and orbital floor resection	ISG-GEIS-OS-24	NO	NO
2	8	F	RE	Proptosis, pain	360° intraorbital extention	2021	Lymphatic malformation	YES				Supraorbital bar + tumor resection	NO	NO	NO
3	1	F	LE	Proptosis	Intraconal mass	2025	Fibrosarcoma	YES	YES	NO	NO	supraorbital bar+ tumor resection	NO	YES	NO
4	5	F	RE	Proptosis,	Intraconal,	2021	Low grade Fibrosarcoma	YES	NO	NO	NO	Resection (anterior cranial approach)	NO	YES	NO
5	9	F	LE	Nasal obstruction, epistaxis, distopia	Etmoidal area, nasal fossa, medial orbital wall, cranial base defect	2025	Radioinduced osteosarcoma	YES	NO	NO	YES	Craniofacial approcah, tumor resection, cranial base reconstruction and titanium mesh	YES	protontherary	NO
6	1	F	LE	Proptosis	intraconal and medial wall	2020	Poorly differentiated neuroblastoma with a low mitosis index	YES	NO	NO	NO	Tumor resection	YES	NO	YES (In RE)
7	9	F	RE	Pain and numbness	maxillary sinus, lateral nasal wall, orbital floor	2025	radioinduced osteosarcoma	YES	NO	NO	YES (BIOMODEL PRINTING)	Tumor removal and orbital floor reconstruction	YES	protontherary	NO
8	5	F	LE	Proptosis, restrictive strabismus, conjunctival venous dilatation	Intraconal and extraconal, extraorbital extension through external canthus	2022	lymphatic malformation	YES	NO	NO	NO	Tumor removal	NO	NO	NO
9	5	F	LE	Lid swelling, proptosis	Infraorbital extended lesion (360°)	2023	Lymphatic malformation	YES	NO	NO	NO	Tumor removal	NO	NO	NO
10	8	M	RE	Proptosis, diplopia	Medial wall, intraconal and postseptal	2021	Neuroendocrine tumor	YES	NO	NO	NO	Tumor excision	IVA	YES	YES
11	1	M	LE	Proptosis	intraconal	2024	orbital encephalocele	YES	NO	NO	NO	Tumor removal and dural reconstruction	NO	NO	NO
12	6	F	RE	Proptosis, extraocular mass, orbital distopia	maxillary sinus, mallar bone, orbital floor	2025	Ossifiyng fibroma	YES	YES	YES	NO	tumor resection and IPS reconstruction	NO	NO	NO

## Data Availability

The datasets presented in this article are not readily available the data are part of an ongoing study. The raw data supporting the conclusions of this article will be made available by the authors on request due to privacy.

## Abbreviations

The following abbreviations are used in this manuscript:

CAS	Computer-assisted surgery
ION	Intraoperative navigation
IPS	Individual Patient Solution
PEEK	Polyether ether ketone
